# Identification of plasma proteins relating to brain neurodegeneration and vascular pathology in cognitively normal individuals

**DOI:** 10.1002/dad2.12240

**Published:** 2021-09-27

**Authors:** Liu Shi, Colin R. Buchanan, Simon R. Cox, Robert F. Hillary, Riccardo E. Marioni, Archie Campbell, Caroline Hayward, Aleks Stolicyn, Heather C. Whalley, Mathew A. Harris, Jennifer Waymont, Gordon Waiter, Ellen Backhouse, Joanna M. Wardlaw, Douglas Steele, Andrew Mcintosh, Simon Lovestone, Noel J. Buckley, Alejo J. Nevado‐Holgado

**Affiliations:** ^1^ Department of Psychiatry University of Oxford Oxford UK; ^2^ Lothian Birth Cohorts Group The University of Edinburgh Edinburgh UK; ^3^ Department of Psychology The University of Edinburgh Edinburgh UK; ^4^ Scottish Imaging Network A Platform for Scientific Excellence (SINAPSE) Collaboration Edinburgh UK; ^5^ Centre for Genomic and Experimental Medicine Institute of Genetics and Molecular Medicine University of Edinburgh Edinburgh UK; ^6^ Medical Research Council Human Genetics Unit Institute of Genetics and Molecular Medicine University of Edinburgh Edinburgh UK; ^7^ Division of Psychiatry University of Edinburgh Edinburgh UK; ^8^ Aberdeen Biomedical Imaging Centre Institute of Medical Sciences University of Aberdeen Aberdeen UK; ^9^ Centre for Clinical Brain Sciences University of Edinburgh Edinburgh UK; ^10^ Dementia Research Institute University of Edinburgh Edinburgh UK; ^11^ Division of Imaging Science and Technology Medical School University of Dundee Scotland UK; ^12^ Centre for Cognitive Ageing and Cognitive Epidemiology University of Edinburgh Edinburgh UK; ^13^ Division of Psychiatry Centre for Clinical Brain Sciences University of Edinburgh Edinburgh UK; ^14^ Janssen R&D London UK

**Keywords:** mediation, neurodegeneration, plasma proteomics, sex‐related difference, vascular damage

## Abstract

**Introduction:**

This study aims to first discover plasma proteomic biomarkers relating to neurodegeneration (N) and vascular (V) damage in cognitively normal individuals and second to discover proteins mediating sex‐related difference in N and V pathology.

**Methods:**

Five thousand and thirty‐two plasma proteins were measured in 1061 cognitively normal individuals (628 females and 433 males), nearly 90% of whom had magnetic resonance imaging measures of hippocampal volume (as N) and white matter hyperintensities (as V).

**Results:**

Differential protein expression analysis and co‐expression network analysis revealed different proteins and modules associated with N and V, respectively. Furthermore, causal mediation analysis revealed four proteins mediated sex‐related difference in N and one protein mediated such difference in V damage.

**Discussion:**

Once validated, the identified proteins could help to select cognitively normal individuals with N and V pathology for Alzheimer's disease clinical trials and provide targets for further mechanistic studies on brain sex differences, leading to sex‐specific therapeutic strategies.

## 1 INTRODUCTION

The National Institute on Aging and Alzheimer's Association (NIA‐AA) have proposed classifying Alzheimer's disease (AD) based on biomarkers of amyloid pathology (A), tau pathology (T), and neurodegeneration (N).^1^ The flexibility of the AT(N) system could be expanded to incorporate new biomarkers that track brain vascular (V) damage, leading to ATV(N).^1^ Both neurodegeneration and vascular pathology can be measured by several different magnetic resonance imaging (MRI) measures. For example, neurodegeneration can be measured by brain atrophy. Multiple lines of evidence showed that hippocampal atrophy is closely associated with AD.^2^, ^3^, ^4^ Vascular pathology can be measured by white matter hyperintensities (WMHs), which have been associated with an increased risk for developing AD and dementia.^5^, ^6^, ^7^

There is increasing evidence to suggest an influence of biological sex on neuroimaging biomarkers in AD pathogenesis. For example, brain atrophy rates for those with mild cognitive impairment (MCI) and AD dementia were faster in females compared to males.^8^, ^9^, ^10^, ^11^ In contrast, increased WMHs led to faster progression to MCI or AD only among males.^12^, ^13^ Although the differences are likely due to sex hormones,^14^ the exact mechanisms that underlie these sex‐related differences are still unclear. Gaining a more detailed picture of the causes of sex‐related differences could yield important clues about the pathophysiology of AD and eventually lead to sex‐specific preventative or therapeutic strategies.

**FIGURE 1:**
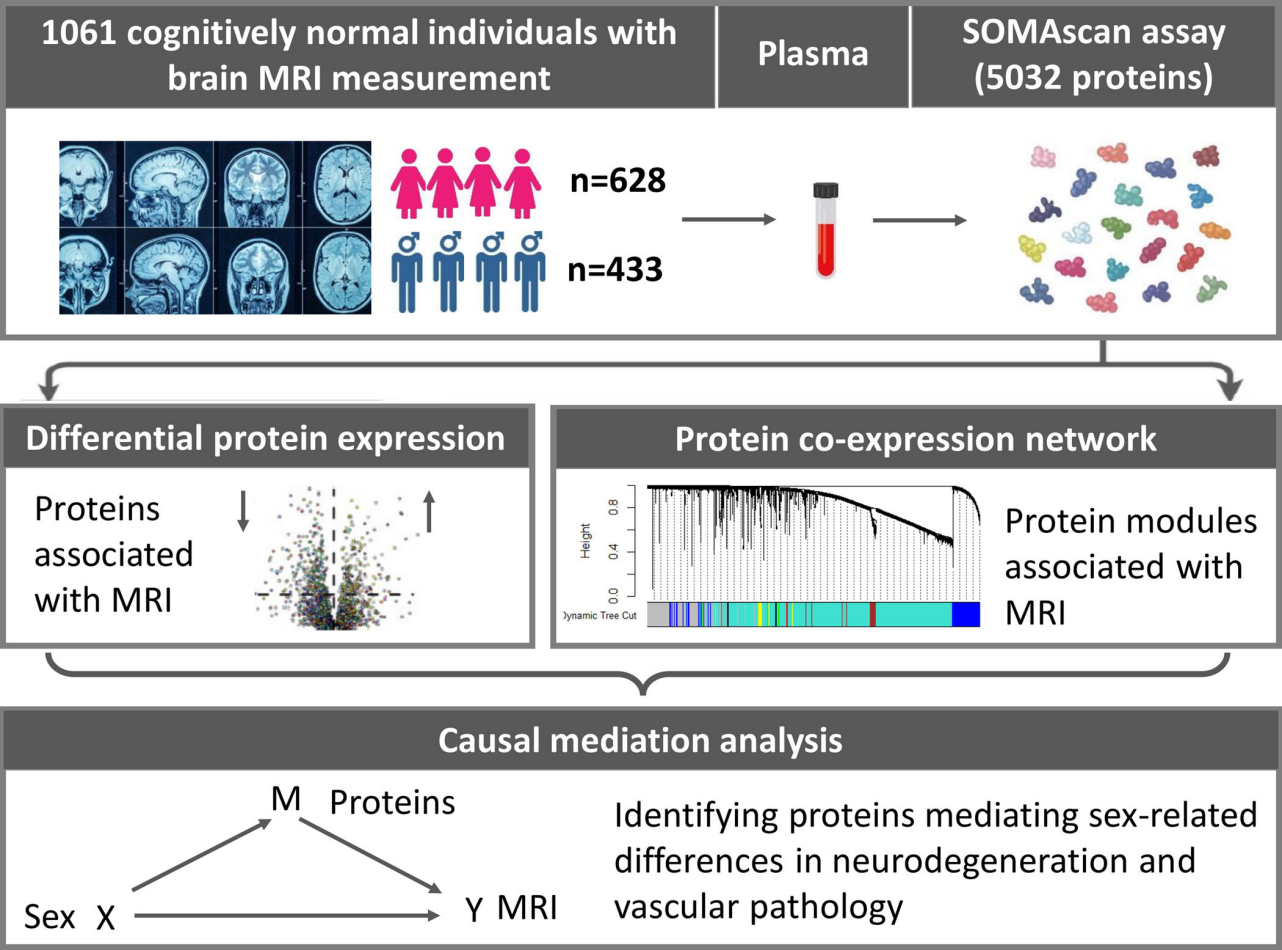
Overview of the study design: 5032 proteins were measured in 1061 cognitively normal individuals who had magnetic resonance imaging (MRI) measurement. Proteome‐wide association study of MRI and protein co‐expression network analysis revealed potential proteins for causal mediation analysis, leading to the finding of proteins mediating sex‐related differences in brain neurodegeneration and vascular damage

Compared to MRI measures, blood‐based biomarkers show promise as a simple and potentially cost‐effective option for the early detection, classification, and monitoring of AD pathology. With this in mind, the present study had two main objectives: first, to identify plasma biomarkers related to neurodegeneration and vascular pathology in cognitively normal individuals; second, to identify proteins mediating sex‐related differences in neurodegeneration and vascular damage. To do this, we used SomaLogic's Somascan assay to measure 5032 proteins in plasma from 1061 cognitively healthy individuals. Using a range of statistical approaches, we identified different proteins associated with markers of neurodegeneration and vascular damage. Furthermore, using causal mediation analysis, we found evidence for several proteins that may mediate sex‐related differences in neurodegeneration and vascular pathology (Figure 1).

## 2 METHODS

### 2.1 Participants

The participants in this study were recruited as part of the Stratifying Resilience and Depression Longitudinally (STRADL) study, which re‐contacted participants from the Generation Scotland: Scottish Family Health Study (GS). The GS study is a large, family‐structured, population‐based cohort study of more than 24,000 individuals from across Scotland. Recruitment took place between 2006 and 2011 with a clinical visit during which detailed health and cognitive test data were collected along with biological samples (blood, urine, saliva). Full details of the STRADL cohort and GS protocol are published elsewhere.^15^, ^16^, ^17^ Briefly, blood was collected by venepuncture into standard polypropylene ethylenediaminetetraacetic acid (EDTA) test tubes followed by centrifugation; the obtained plasma was aliquoted in 500 μL aliquots, and stored at −20°C for future analyses.^17^ Ethical approval for STRADL was formally obtained from the National Health Service Tayside committee and all participants provided their written informed consent.

**RESEARCH IN CONTEXT**

1. **Systematic review**: Plasma proteins are studied as candidate biomarkers to predict brain neurodegeneration and vascular pathology in Alzheimer's disease (AD), while few studies focus on prodromal stage of AD. Furthermore, there is increasing evidence to suggest brain sex differences in AD pathogenesis, while the exact mechanisms that underlie these sex‐related differences are still unclear.
2. **Interpretation**: Our findings offer new insights into changes in individual proteins and protein networks linked to neurodegeneration and vascular pathology in the prodromal stage of AD. Furthermore, we identified several plasma proteins mediated sex‐related differences in brain neurodegeneration and vascular damage. Our study is the largest plasma proteomic study in prodromal AD in terms of the number of proteins assayed and sample size, to our knowledge.
3. **Future directions**: It suggests that blood proteins can predict brain neurodegeneration and vascular pathology in the prodromal stage of AD. Furthermore, the nominated proteins are tractable targets for further mechanistic studies on brain sex differences, with potential to lead to more effective sex‐specific preventative or therapeutic strategies.

We selected a total of 1061 cognitively normal individuals including 628 females and 433 males from STRADL study. Plasma and general demographic information were available for all subjects (including family history of AD and apolipoprotein E [*APOE*] ε4 genotype data). Four cognitive tests covering multiple cognitive domains were also assessed for each subject. These tests included digit symbol, verbal fluency, Mill Hill vocabulary scale, and Wechsler memory test.^15^ A general cognitive ability score was generated via extracting the first unrotated component from a principal component analysis of the four tests. Brain structural MRI scans were available for 927 participants to obtain hippocampal volume. Visually inspecting and rating fluid‐attenuated inversion recovery (FLAIR) scans were available for 940 subjects to obtain Fazekas score, an index of WMHs.

### 2.2 Plasma analyses

Plasma proteins were measured using the SOMAscan assay platform (SomaLogic Inc.). SOMAscan is an aptamer‐based assay allowing for the simultaneous measurement and quantification of, in the version used here, 5032 proteins. The assay uses chemically modified nucleotides to transform a protein signal into a nucleotide signal that can be quantified using relative fluorescence on microarrays.^18^ Raw data processing and initial quality control (QC) led to 4235 proteins for final analysis. The abundance of each protein was log‐transformed, then the effects of sample collection site and plasma storage time on proteins were removed by linear regression and the residuals were used for all subsequent analyses.

### 2.3 MRI acquisition and analyses

Participants were scanned at two centers: the Ninewells Hospital in Dundee and at the Aberdeen Royal Infirmary in Aberdeen. Participants in Dundee were scanned using a Siemens 3T Prisma‐FIT (Siemens Healthineers) with a 20‐channel head and neck coil and a back‐facing mirror (software version VE11, gradient with max amplitude 80 mT/m and maximum slew rate 200 T/m/s). In Aberdeen, participants were imaged on a 3 T Philips Achieva TX series MRI system (Philips Healthcare) with a 32‐channel phased‐array head coil with a back‐facing mirror (software version 5.1.7; gradients with maximum amplitude 80 mT/m and maximum slew rate 100). Both study centers followed the same protocol including structural sequences.^17^ 3T MRI scans were anonymized at the time of acquisition and scanning site was included as a covariate in statistical analyses. Full details of the imaging sequences and parameters are published elsewhere.^19^

#### 2.3.1 Hippocampal volume

T1 structural measures were processed using FreeSurfer version 5.3^20^ to quantify the volumes of 14 subcortical structures as well as the volumes, surface area, and thickness of 34 cortical regions per hemisphere according to the Desikan‐Killany atlas.^21^ Full details of the FreeSurfer QC steps are published elsewhere.^19^ Measures of thickness, surface area, and volume were derived for each of the 68 cortical regions. The volumes of 14 subcortical structures—left and right accumbens area, amygdala, caudate nucleus, hippocampus, pallidum, putamen, and thalamus—were also extracted from FreeSurfer output. Global measures of cortical volume, surface area, and thickness were also derived, as well as five summed lobar measures (frontal, parietal, temporal, occipital, and cingulate). The number of QC edits made per individual were recorded to use as a covariate in statistical analyses. Among all those measures, only hippocampal volume data are used in this study as it is highly related to AD.^2^, ^3^, ^4^

#### 2.3.2 WMHs—Fazekas scores

WMHs was calculated using FLAIR scan files. It was defined as punctuate, focal, or diffuse lesions in the deep or periventricular white matter, basal ganglia, or brainstem, visible as areas of hyperintensity on FLAIR images with respect to normal‐appearing white or gray matter. Severity of WMHs was graded according to the Fazekas scale,^22^ which distinguishes periventricular WMHs and deep WMHs and grades them from 0 (absent) to 3 (severe). A score of 1 is defined as caps or pencil thin lining (periventricular WMHs, Figure S1A1 in supporting information) or punctuate foci (deep WMHs, Figure S1B1); a score of 2 is defined as a smooth halo (periventricular WMHs, Figure S1A2) or beginning to confluence (deep WMHs, Figure S1B2) and a score of 3 is defined as irregular periventricular signal extending into the deep white matter (periventricular WMHs, Figure S1A3) or large confluent areas (deep WMHs, Figure S1B3). We used the total scores in this study by summing the periventricular and deep white matter scores.

### 2.4 Statistical analysis

Statistical analyses were completed using R (version 3.3.2). To compare baseline cohort characteristics between males and females, we used Mann‐Whitney U tests and Chi‐square tests to compare continuous and binary variables, respectively.

#### 2.4.1 Protein differential expression analysis (DEA)

We used partial Spearman correlation to test the association of proteins with hippocampal volume and WMHs (Fazekas scores), adjusting for age, sex, and *APOE* ε4 genotype. Additionally, imaging batch, number of image edits per individual, assessment center, and standardized intracranial volume (ICV) were set as covariates for hippocampal volume analyses. We performed such analyses in all individuals as well as in subgroups stratified by sex. *P* values were corrected using a false discovery rate (FDR) for multiple testing. Proteins that were differentially expressed at a nominal significance level of *P* < .05 were included in pathway analysis using WebGestalt software (http://www.webgestalt.org/). Briefly, differentially expressed proteins were included as the “protein list” and all proteins measured by SOMAscan assay were used as “background.” This enrichment analysis was performed on the Kyoto Encyclopedia of Genes and Genomes (KEGG) database.

#### 2.4.2 Weighted gene correlation network analysis (WGCNA)

We first used the R package WGCNA^23^ to construct a co‐expression network from the proteins. The effects of age and sex on proteins were adjusted for by linear regression and the resulting residuals were used for analysis. WGCNA clustering is based on calculating correlations between paired variables, soft‐threshold transforming them with a power function (cor^β^), and using the result as adjacency matrix between variables. The final step applies hierarchical clustering to this adjacency matrix. We applied this algorithm with default parameters, except for the following settings: soft threshold power beta = 4, minimum module size = 10 proteins, merge cut height = 0.2. The resulting modules or groups of co‐expressed proteins were used to calculate module eigenproteins. The eigenprotein‐based connectivity (kME) was used to represent the strength of a protein's correlation with other protein module members. Proteins with high intramodular kME in the top 90th percentile within a module were considered hub proteins. KEGG pathway enrichment was performed for each module using WebGestalt software as described previously. Briefly, proteins within a module were assembled into a “protein list” and all proteins were used as “background.”

We then calculated the association of eigenproteins with hippocampal volume and WMHs in all individuals as well as in only female and male individuals, adjusting for age, sex, and *APOE* ε4 genotype. The additional covariates for hippocampal volume were imaging batch, number of image edits per individual, assessment center, and ICV. The *P* values were corrected with FDR and corrected *P* values were presented in a heatmap. Furthermore, we used Student's t‐test to assess pairwise difference of eigenproteins among different AD risk groups. The AD risk was decided by both family history and *APOE* ε4 genotype. In detail, individuals without family history (self‐reported as no parent with AD) and without a copy of the *APOE* ε4 allele were categorized as low risk; individuals with either family history or *APOE* ε4 were defined as being at medium risk; individuals with both were classified as high risk.

#### 2.4.3 Causal mediation analysis

We used R package regmedint^24^ to investigate how the relationship between an exposure variable and an outcome variable relate to a third intermediate variable, namely the mediator. Here, we wanted to test if proteins (as mediator, M) mediate the relationship between sex (as exposure, X) and MRI measurement (as outcome, Y). A mediator needs to meet the following three criteria^25^ (Figure S2A in supporting information): (1) A change in levels of the exposure variable significantly affects the changes in the outcome (i.e., total effect of X on Y is significant). (2) There is a significant relationship between the mediator and the outcome (i.e., Path from M to Y). (3) A change in levels of the exposure variable significantly affects the changes in the mediator (i.e., Path from X to M).

There was a significant difference between females and males in terms of hippocampal volume and WMHs after adjusting for covariates (Figure S2B and C; criteria 1). For mediators, we selected proteins significantly associated with hippocampal volume or WMHs (criteria 2). We also checked that these proteins are significantly expressed between females and males (criteria 3). We reported those proteins whose natural indirect effect is significant and the direction is consistent with natural direct effect.

## 3 RESULTS

### 3.1 Subject demographics

Demographic information of subjects is shown in Table 1. The male group was slightly older than the female group. No significant difference was observed in the distribution of *APOE* ε4 carriers, education, and self‐reported family history (mother or father reported as having AD). In terms of MRI measures, females had a smaller hippocampal volume than males on average, while no difference was observed in WMHs.

**TABLE 1:** Demographics of participants included in the analysis by sex

Characteristics	Female (n = 628)	Male (n = 433)	*P* value
Age mean (SD), y	59.3 ± 9.7	60.7 ± 9.3	0.02
*APOE* ε4+ N (%)	170 (27%)	118 (27%)	0.99
Education mean (SD), y	4.5 (1.5)	4.5 (1.6)	0.83
Mother with AD	46 (7.3%)	27 (6.2%)	0.57
Father with AD	24 (3.8%)	15 (3.5%)	0.89
Hippocampal volume (SD) in mm^3^	8155 (744)	8659 (936)	<0.001
WMHs (SD)	2.27 (0.92)	2.21 (0.88)	0.35

Abbreviations: AD, Alzheimer's disease; *APOE*, apolipoprotein E; SD, standard deviation; WMHs, white matter hyperintensities.

Note: Percentage of cases is shown in parentheses for *APOE* ε4 carriers and mother or father with AD.

### 3.2 Proteins significantly associated with hippocampal volume and WMHs

**FIGURE 2:**
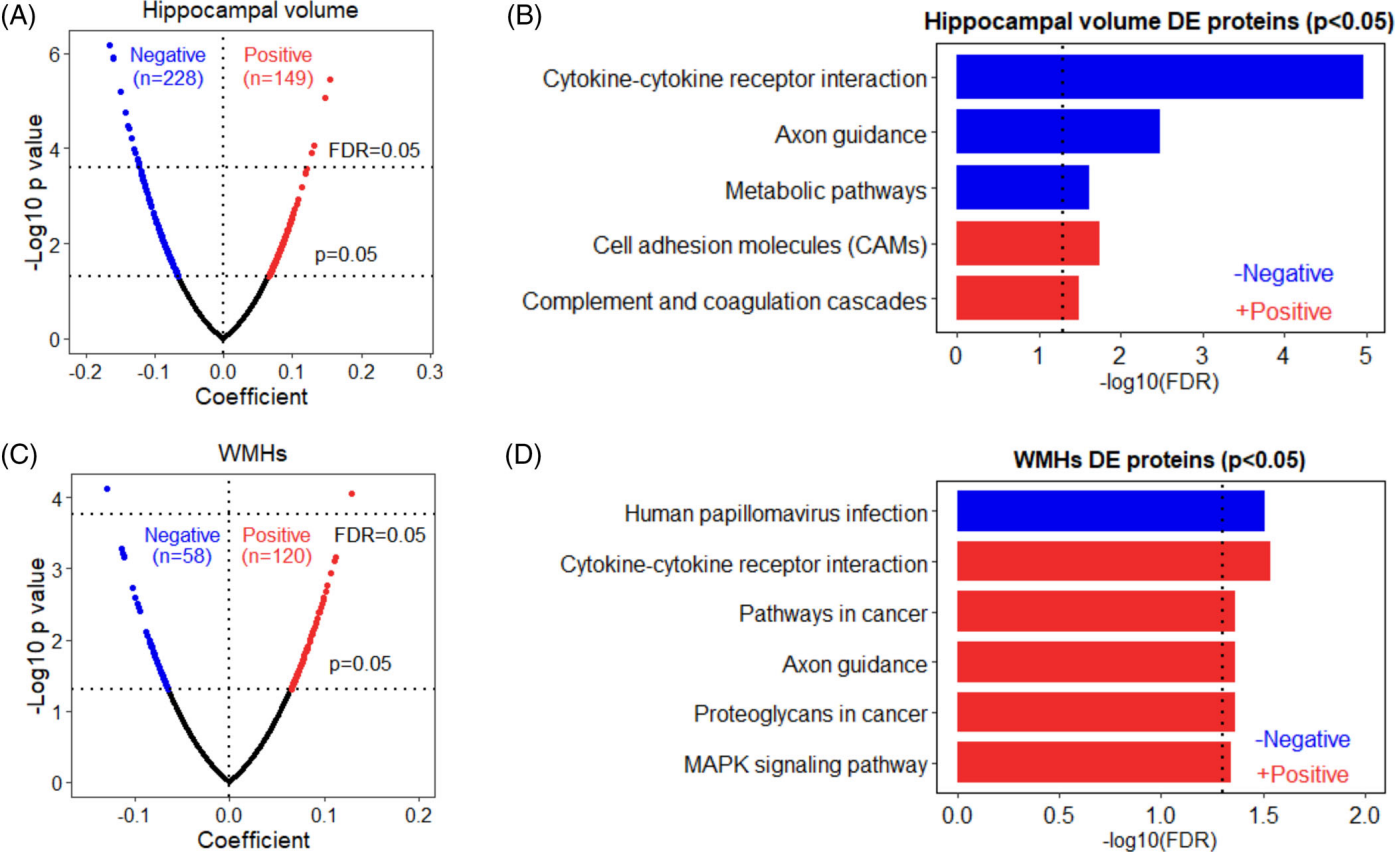
Volcano plot of proteins associated with (A) hippocampal volume and (C) white matter hyperintensities (WMHs). Those proteins with significantly negative correlations (*P *< .05) are shown in blue, while the proteins with significantly positive correlations (*P *< .05) are noted in red. Enriched Kyoto Encyclopedia of Genes and Genomes pathways of proteins with significantly negative (blue) and positive (red) associations with (B) hippocampal volume and (D) WMHs. DE, differential expression; FDR, false discovery rate

Using partial Spearman correlation, we found nominally significant associations (*P *< .05) between 377 proteins and hippocampal volume in all individuals. Seventeen of these were significant after FDR correction for multiple testing (FDR *P* < .05; Figure 2A, Table S1 in supporting information). KEGG pathway analysis of the 228 proteins with significantly negative associations (*P *< .05) revealed three pathways including cytokine–cytokine receptor interaction, axon guidance, and metabolic pathways (Figure 2B). Conversely, the 149 proteins with significantly positive associations (*P *< .05) indicated two pathways that were cell adhesion molecules (CAMs) and complement and coagulation cascades (Figure 2B). Stratifying by sex, we found 328 proteins showed nominally significance in females and 2 of them were significant after FDR correction. In contrast, 172 proteins showed nominally significance in males while no proteins were significant after FDR correction.

For WMHs, 178 proteins showed nominal significance and 2 of them remained significant after FDR correction in all individuals (Figure 2C, Table S1). KEGG pathway analysis of the proteins with significantly negative and positive associations (*P *< .05) revealed one and five enriched pathways, respectively (Figure 2D). None of the proteins were significant after FDR correction in either females or males. Of the 17 hippocampal volume‐related and 2 WMH‐related proteins (FDR *P *< .05), 8 of them were significantly associated with general cognitive score (Figure S3 in supporting information).

### 3.3 Plasma protein co‐expression network analysis reveals modules linked to hippocampal volume and WMHs

**FIGURE 3:**
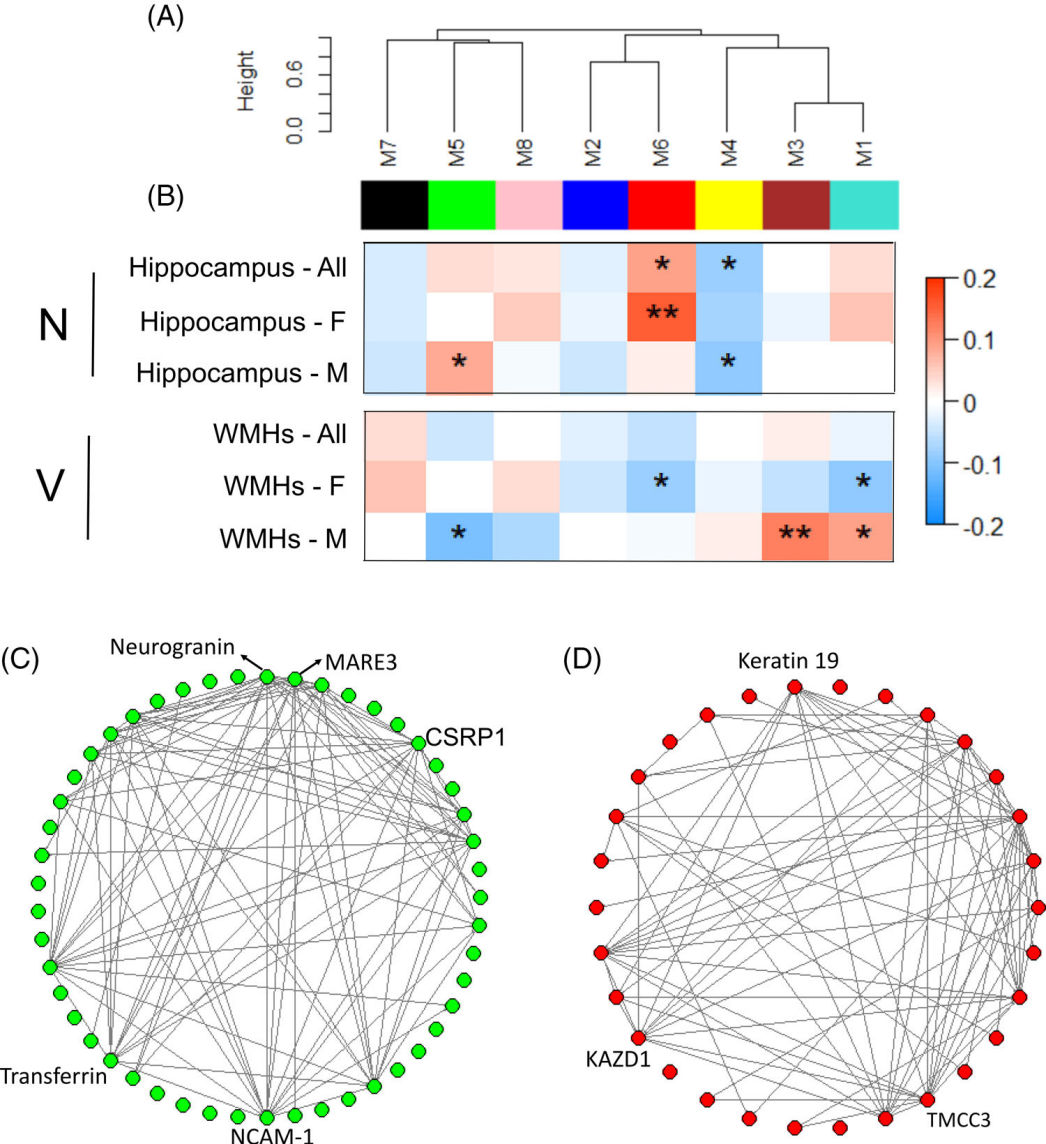
Protein modules correlating to hippocampal volume and white matter hyperintensities (WMHs). A, Weighted gene correlation network analysis (WGCNA) of the plasma proteome. This algorithm generated eight modules (*M*) of co‐expressed proteins. Modules are clustered in the network dendrogram based on their relatedness. B, Analysis of the association of modules with hippocampal volume and WMHs. * and ** denote significant correlations *P* < 0.05 and *P *< 0.001 after false discovery rate (FDR) correction, respectively. (C) and (D) Hub proteins noted within the *M5 green* and *M6 red* modules, respectively. F, female; M, male; N, neurodegeneration; V, vascular damage

We first performed a network‐based analysis of the plasma proteome using WGCNA. We found eight modules (*M*) of co‐expressed proteins and ranked them based on size from largest (*M1 turquoise* module; n = 2694 proteins) to smallest (*M8 pink* module; n = 13 proteins; Table S2 in supporting information). Figure 3A shows the clustering of these modules’ concordance according to similarities in expression patterns. We further investigated the biological significance of proteins in each module and found that the modules were enriched with various pathways after FDR correction (Table S3 in supporting information), such as renal cell carcinoma (*M1 turquoise* module), cytokine–cytokine receptor interaction (*M2 blue* module), metabolism of xenobiotics by cytochrome P450 (*M4 yellow* module), complement and coagulation cascades (*M5 green* module), cholesterol metabolism (*M6 red* module), and pancreatic secretion (*M8 pink* module).

We then assessed the module correlations to hippocampal volume and WMHs in all individuals as well as only in females and males. As shown in Figure 3B, across all individuals and after FDR correction, the *M4 yellow* and *M6 red* modules had a negative and positive correlation with hippocampal volume, respectively. Furthermore, such correlations remained significant in males and females and their direction of association were consistent. In addition, the *M5 green* module was positively correlated with hippocampal volume in males.

For WMHs, none of the modules passed FDR in all individuals. However, in females, the *M1 turquoise* and *M6 red* modules had negative correlations with WMHs. In males, the *M1 turquoise* and *M3 brown* modules were positively associated with WMHs while the *M5 green* module was negatively associated with WMHs (Figure 3B). Furthermore, modules that were positively correlated with hippocampal volume were negatively associated with WMHs such as the *M5 green* and *M6 red* modules, indicating the consistency between such associations because hippocampal atrophy and larger WMHs are associated with increased AD risk. Figures 3C and D showed the hub proteins within the *M5 green* and *M6 red* modules, respectively.

### 3.4 Correlation of protein networks with cognitive test scores and AD risk

**FIGURE 4:**
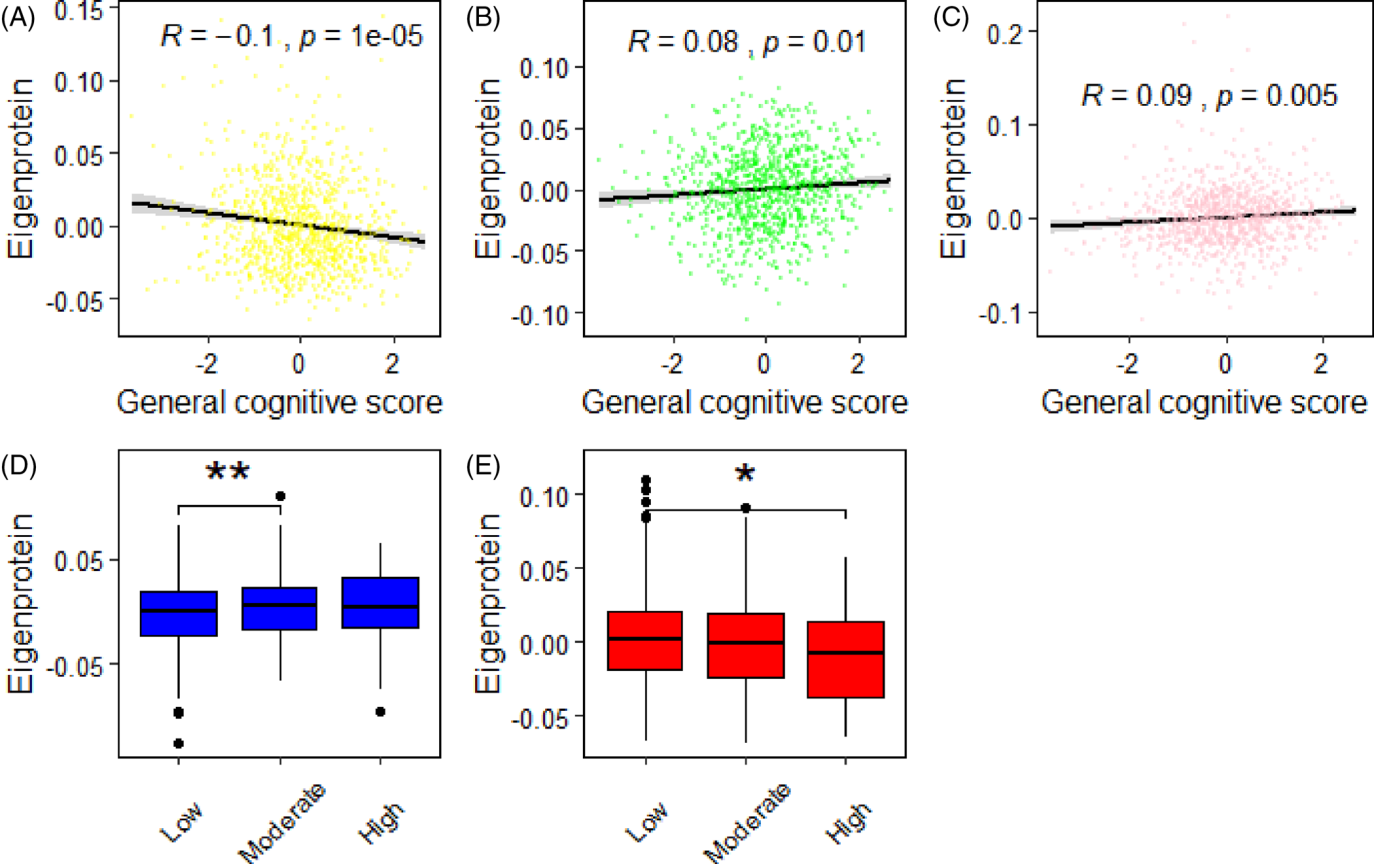
Protein modules associations with (A‐C) general cognitive score and (D‐E) Alzheimer's disease risk

We further investigated the module correlations to cognitive test measures and AD risk. We found that the *M4 yellow* and *M8 pink* modules had negative correlations and *M5 green* module had a positive correlation with general cognitive score after FDR correction (Figure 4A‐C). These results are in concordance with MRI correlations as the *M4 yellow* and *M5 green* modules had negative and positive correlations with hippocampal volume, respectively. The AD risk was decided by both family history and *APOE* ε4 genotype as described previously, leading to 662 low risk, 315 moderate risk, and 32 high risk individuals. We found that the *M2 blue* and *M6 red* modules showed significant increase and decrease in higher risk compared to low‐risk individuals, respectively (Figure 4D and E). Of these, the *M6 red* module is in concordance with MRI correlations as it had positive and negative correlations with hippocampal volume and WMHs, respectively.

### 3.5 Causal mediation analysis reveals proteins mediating brain sex differences

We selected proteins obtained from DEA for further causal mediation analysis. Overall, we considered 17 hippocampal volume‐related and two WMH‐related proteins (FDR *P *< .05). Of the 17 proteins, 4 of them showed significant natural indirect effect with hippocampal volume (Table 2), indicating they mediated sex‐related differences in brain neurodegeneration. Furthermore, two of them were in the *M5 green* module, which had positive correlations with hippocampal volume only in males (Figure 3B) and NCAM‐1 was the hub protein in the *M5 green* module (Figure 3C), further indicating the consistency across DEA and co‐expression network analysis. For WMHs, one protein (Apo B) showed a significant natural indirect effect (Table 2), meaning it mediated sex‐related difference in vascular damage. This protein is in the *M6 red* module, which had a negative correlation with WMHs only in females (Figure 3B).

**TABLE 2:** Mediation of the association between sex and MRI brain variables by proteins

MRI measures	Protein	Module	Effect estimate [95% CI]	*P* value
Hippocampal volume	NCAM‐1	Green	0.04 [0.02, 0.07]	0.002
	EFEMP1	Green	0.02 [0.003, 0.05]	0.03
	R‐spondin‐1	Turquoise	0.03 [0.01, 0.06]	0.008
	FSTL3	Turquoise	0.03 [0.002, 0.05]	0.03
WMHs	Apo B	Red	–0.03 [–0.05, –0.01]	0.008

Abbreviations: CI, confidence interval; MRI, magnetic resonance imaging; WMHs, white matter hyperintensities.

## 4 DISCUSSION

In this study, we used SOMAscan to measure 5032 plasma proteins from 1061 cognitively normal individuals (female n = 628 and male n = 433). We performed both proteome‐wide DEA and protein co‐expression network analysis to gain insights into changes in individual proteins as well as networks of proteins relating to neurodegeneration and vascular markers in cognitively healthy individuals. We identified different proteins and modules associated with markers of neurodegeneration and vascular pathology, respectively. Using causal mediation analysis, we further confirmed that four proteins mediated sex‐related difference in brain neurodegeneration and one protein mediated such difference in vascular damage. Of the five proteins, three of them were in the modules that were associated with neurodegeneration or vascular damage only in male or female and one protein (NCAM‐1) was the hub protein in the module, suggesting that our findings are robust across multiple computational analyses. In addition, most neurodegeneration‐ and vascular pathology–related proteins and modules were also associated with cognitive score and their direction of change was in concordance with MRI correlations.

It is important to identify blood‐based biomarkers relating to neurodegeneration, particularly in the preclinical stage of AD. Most clinical trials for AD have been unsuccessful to date.^26^ The failure of such trials was partially caused by the fact that participants enrolled in such trials were relatively late in the disease process. Targeting treatment to earlier pre‐symptomatic or prodromal stages of the disease might have more success.^27^ With this in mind we sought to identify blood biomarkers relating to neurodegeneration in cognitively normal individuals collected from a population‐based cohort that resembles the real‐world situation of participants’ recruitment in clinical trials. We identified 17 proteins that were significant after FDR correction for multiple testing in the full cohort; two proteins were FDR significant in females compared to no proteins in males. This might be because brain atrophy is small in cognitively normal individuals and that the subgroup analysis is too small to detect such differences. Nevertheless, because these proteins were associated with neurodegeneration in the preclinical stage, they could potentially help when recruiting cognitively normal individuals with neurodegeneration for clinical trials with further validation.

The importance of WMHs in AD is increasingly recognized as they are related to risk for developing AD.^5^, ^6^, ^7^ Some studies showed that there is an association between WMHs and AD pathological hallmarks such as amyloid beta (Aβ) plaques, tau, and neurodegeneration.^28^, ^29^, ^30^ Here, we only found two proteins that were associated with WMHs after FDR correction for multiple testing. This might be because the participants recruited in our study were relatively young whereas WMHs tend to be observed with aging.^31^, ^32^ Current treatment of WMHs includes pharmacological approaches (i.e., blood pressure medications and statins)^33^, ^34^, ^35^ as well as nonpharmacological approaches such as lifestyle modifications and risk factor management.^36^, ^37^, ^38^ Given the associations between WMHs and vascular risk factors, it is imperative to target vascular health throughout the life course as a prevention strategy. The proteins identified here could potentially help to select cognitively normal individuals with vascular pathology at a very early stage as well as to monitor the intervention outcomes throughout the life course.

Current findings on the associations of AD hallmarks in plasma with neurodegeneration and vascular pathology have generated new enthusiasm. For example, various studies found that neurofilament light chain (NfL) was closely associated with neurodegeneration.^39^, ^40^, ^41^ Furthermore, NfL was also associated with vascular pathology in an age‐dependent manner.^42^ Another marker, phosphorylated tau (p‐tau)181 was found to be a good marker for predicting and monitoring neurodegeneration.^43^, ^44^ These two biomarkers are well recognized as they have been validated in multiple cohorts across different laboratories. In comparison, the identified biomarkers from our study need further validation in independent longitudinal cohorts. Furthermore, it is important to conduct head‐to‐head comparison studies in the future not only to compare the performance of our identified biomarkers to both NfL and p‐tau181 but also to check whether they could add extra value on top of these two well‐recognized biomarkers.

The increasing recognition of sex differences in the brain and AD risk has highlighted the urgent need for biomarkers that more comprehensively reflect the complex mechanisms underlying these differences.^45^, ^46^ Examination of these differences may shed light on the pathophysiology of AD that differs between the sexes and ultimately lead to more effective interventions and precision medicine. Here, we reported a study that characterized plasma proteins mediating brain sex differences in cognitively normal individuals in the largest sample to date. Using a range of statistical approaches, we identified four proteins that mediated sex‐related difference in brain neurodegeneration and one protein in vascular damage.

Of the five proteins, neural cell adhesion molecule (NCAM) is a part of a family of cell‐surface glycoproteins that play= key roles in normal brain development, including axonal/dendritic growth and branching, and synaptic plasticity.^47^ The levels of NCAM‐1 have been shown to alter in AD patients’ blood, cerebrospinal fluid (CSF), and brain tissue.^48^, ^49^, ^50^ Furthermore, it interacted with amyloid precursor protein (APP) and promoted neurite outgrowth, indicating that it could be a potential therapeutic target for AD treatment.^51^, ^52^ Apolipoprotein B (Apo B) forms the primary protein component of atherogenic lipoprotein particles. Lower levels of this protein was associated with better maintenance of cognitive abilities.^53^ Furthermore, it was highly associated with Aβ and tau pathology in subjective cognitive decline individuals, indicating that Apo B may be a potential biomarker for the preclinical stage of AD.^54^ R‐spondin‐1 is encoded by the *RSPO1* gene on chromosome 1 and expressed in the central nervous system during development.^55^ This protein has been shown to increase in CSF of presymptomatic and affected persons carrying familial AD mutations^56^ as well as to reduce Aβ and reverse cognitive impairment in AD mouse model.^57^ The other two proteins were EGF‐containing fibulin‐like extracellular matrix protein 1 (EFEMP1), a member of the fibulin family that mediates cell–cell and cell–matrix interactions^58^; follistatin‐related protein 3 (FSTL3), an important physiological regulator of activin; and other TGFβ superfamily members.^59^ These findings not only confirm the role of previously reported proteins in AD, but also identify new biomarkers relating to early neurodegeneration pathology such as EFEMP1 and FSTL3. Furthermore, these findings provide tractable targets for further mechanistic studies of sex‐related neurodegeneration and vascular pathology in the preclinical stage of AD, eventually leading to sex‐specific preventative or therapeutic strategies.

Pathway analysis of hippocampal volume‐related and WMH‐related proteins revealed five and six significantly enriched pathways, respectively. Some of them have been reported being associated with AD such as axon guidance,^60^ MAPK signaling pathway,^61^ and cell adhesion molecules (CAMs),^62^ further demonstrating the relatedness of these proteins with AD. Furthermore, most neurodegeneration and vascular damage‐related proteins and protein modules were associated with cognitive score and their direction of association were in concordance with brain MRI correlations. Various studies have reported that brain atrophy and increased WMHs lead to cognitive decline.^63^, ^64^, ^65^, ^66^, ^67^ Our finding further demonstrated that proteins relating to brain pathology were also associated with lower cognitive test scores.

There are three limitations for our study. First, the population in this study is of European ancestry, predominantly of Scottish ancestry, so validation in independent cohorts and particularly in other ethnic groups is needed to see if the results are generalizable. Second, our study is cross‐sectional and longitudinal studies are required to determine the role of nominated proteins in sex differences in brain pathology and risk of AD. Third, although the individuals in this study are well characterized on cognition, *APOE* ε4 genotype, and family history, there is a lack of Aβ plaques and tau tangles to confirm the stage of AD. Therefore, the terminology of preclinical AD needs to be interpreted with caution. Further studies on individuals defined by both neuropathology and clinical symptoms are needed to confirm the results.

Despite this, our study is the largest we are aware of to report plasma biomarkers indicative of both neurodegeneration and vascular damage in cognitively normal individuals in terms of the number of proteins assayed as well as sample size. By applying different statistical approaches, we identified individual proteins and protein networks linked to N and V in the poorly understood preclinical stage of AD. Furthermore, we demonstrated that four proteins mediated sex‐related differences in brain neurodegeneration and one protein in vascular damage. These nominated proteins can not only serve as biomarkers to help recruit cognitively normal individuals with neurodegeneration and vascular damage for clinical trials but also as predictive biomarkers to monitor possible intervention outcomes. Furthermore, these proteins provide tractable targets for further mechanistic studies of sex differences in brain pathology and AD risk, with potential to lead to more effective sex‐specific preventative or therapeutic strategies.

## CONFLICTS OF INTEREST

S.R.C received payment from the Society of Biological Psychiatry (plenary at SOBP2021). R.E.M. is an advisor to the Epigenetic Clock Development Foundation and has received a speaker fee from Illumina. A.C. is member of Edinburgh MVM Research Ethics Committee. J.M.W is involved in European Stroke Organisation Guideline on Covert Small Vessel Disease 2021 and European Stroke Organisation Chair of Conference Planning Group 2021 and 2022. D.S. helped to set up CAPE study. A.M. received speaker fees from Janssen and Illumina. S.L. is an employee of Janssen Medical UK and Co‐founder of Akrivia Health Ltd. He is also named as an inventor on biomarker intellectual property protected by Proteome Sciences and Kings College London unrelated to the current study and within the past 5 years has advised for Optum labs, Merck, SomaLogic, and been the recipient of funding from AstraZeneca and other companies via the IMI funding scheme. N.B. is a member of Mehta Family Centre for Engineering in Medicine, Kanpur, India as well as the editorial board of *Stem Cells*. A.N.H. is the main PI of a project funded by J&J, and another projects funded by GSK, all unrelated to this study. The remaining authors declare that the research was conducted in the absence of any commercial or financial relationships that could be construed as a potential conflict of interest.

## ETHICS STATEMENT

Ethical approval for the GS:SFHS study was obtained from the Tayside Committee on Medical Research Ethics (on behalf of the National Health Service).

## Supporting information

Supplementary information

## Data Availability

Access to and use of GS and STRADL data must be approved by the GS Access Committee under the terms of consent. Full details of the application process can be found at www.generationscotland.org.
