# Supplementary material for: Identification of plasma proteins relating to brain neurodegeneration and vascular pathology in cognitively normal individuals
Source: Alzheimers Dement (Amst). 2021 Sep 27;13(1):e12240. doi: 10.1002/dad2.12240 (PMC8474123; doi:10.1002/dad2.12240)
Supplement: Supplementary file 1 — Supplementary information [file DAD2-13-e12240-s001.docx]

| 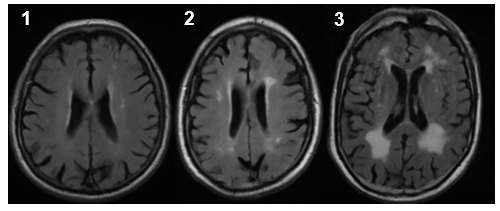 | 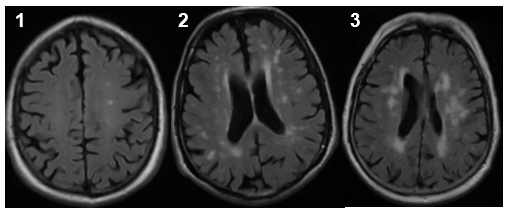 |
| --- | --- |
| **A** | **B** |

**Figure S1** Axial FLAIR images showing examples of the grading of white matter hyperintensities using the Fazekas scale. Examples are presented for scores 1-3 for (A) periventricular white matter hyperintensities (B) deep white matter hyperintensities. FLAIR: Fluid-attenuated inversion recovery.

**
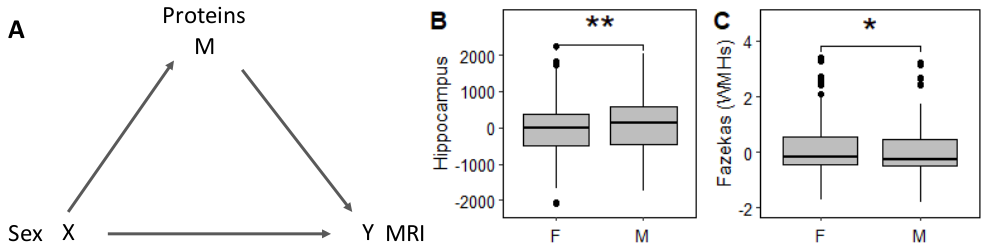
**

**Figure S2** A, Mediation model; B and C represent hippocampal volume and WMHs with sex respectively. WMHs, white matter hyperintensities; F, female; M, male.

| **UniProt** | **beta** | ***p value*** | **FDR *p* value** | **Protein Name** | **Full Name** |
| --- | --- | --- | --- | --- | --- |
| **Hippocampal volume** | | | | | |
| O95633 | -0.165 | 0.000 | 0.002 | FSTL3 | Follistatin-related protein 3 |
| Q9UBR2 | -0.161 | 0.000 | 0.002 | CATZ | Cathepsin Z |
| P09529 | -0.161 | 0.000 | 0.002 | Inhibin bB chain | Inhibin beta B chain |
| P01215 | 0.154 | 0.000 | 0.004 | Glycoprotein hormones a-chain | Glycoprotein hormones alpha chain |
| Q9BUD6 | -0.150 | 0.000 | 0.005 | SPON2 | Spondin-2 |
| P13591 | 0.148 | 0.000 | 0.006 | NCAM1 | Neural cell adhesion molecule 1, 120 kDa isoform |
| P39900 | -0.143 | 0.000 | 0.011 | MMP-12 | Macrophage metalloelastase |
| P07686 | -0.138 | 0.000 | 0.017 | Hexosaminidase B | Beta-hexosaminidase subunit beta |
| P17900 | -0.137 | 0.000 | 0.017 | SAP3 | Ganglioside GM2 activator |
| Q2MKA7 | -0.133 | 0.000 | 0.026 | R-spondin-1 | R-spondin-1 |
| O60476 | 0.131 | 0.000 | 0.033 | MA1A2 | Mannosyl-oligosaccharide 1,2-alpha-mannosidase IB |
| P15586 | -0.129 | 0.000 | 0.036 | GNS | N-acetylglucosamine-6-sulfatase |
| P07998 | -0.128 | 0.000 | 0.038 | RNase 1 | Ribonuclease pancreatic |
| O14791 | 0.128 | 0.000 | 0.038 | Apo L1 | Apolipoprotein L1 |
| P20333 | -0.125 | 0.000 | 0.046 | TNF sR-II | Tumor necrosis factor receptor superfamily member 1B |
| Q92626 | -0.125 | 0.000 | 0.046 | PXDN | Peroxidasin homolog |
| Q12805 | -0.124 | 0.000 | 0.050 | EFEMP1 | EGF-containing fibulin-like extracellular matrix protein 1 |
| **WMHs** | | | | | |
| P04114 | -0.131 | 0.000 | 0.046 | Apo B | Apolipoprotein B |
| Q96D42 | 0.129 | 0.000 | 0.046 | TIM-1 | Hepatitis A virus cellular receptor 1 |

**Table S1** Proteins remained significant after false discovery rate (FDR) correction from partial correlation with hippocampal volume and WMHs. WMHs, white matter hyperintensities.

| 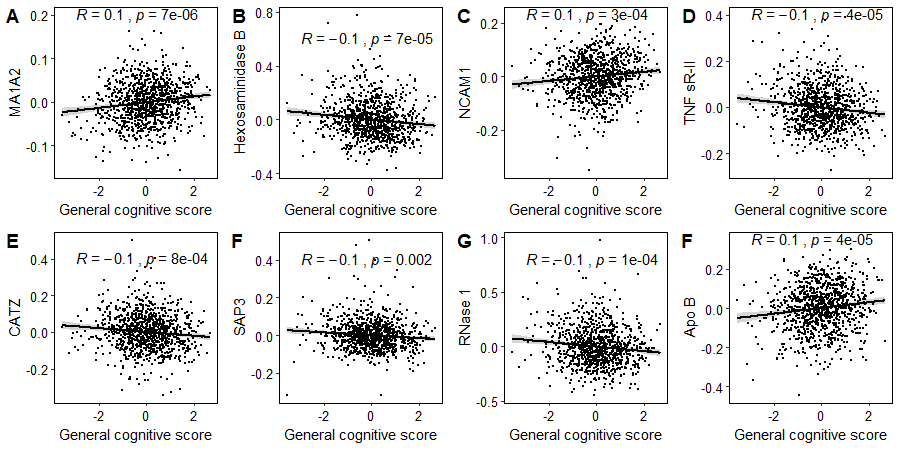 |
| --- |

**Figure S3** Correlation of general cognitive score with (A-G) hippocampal volume related proteins and (F) white matter hyperintensities related protein.

|  | ***M1 turquoise*** | ***M2 blue*** | ***M3 brown*** | ***M4 yellow*** | ***M5 green*** | ***M6 red*** | ***M7 black*** | ***M8 pink*** |
| --- | --- | --- | --- | --- | --- | --- | --- | --- |
| Number of proteins | 2694 | 580 | 112 | 74 | 49 | 30 | 19 | 13 |

**Table S2** The number of proteins in the eight modules (M).

| **Modules** | **Pathways** | **P Value** | **FDR** |
| --- | --- | --- | --- |
| *M1 Turquoise* | Renal cell carcinoma | 0.000 | 0.027 |
|  | Apoptosis | 0.001 | 0.027 |
|  | Insulin signaling pathway | 0.001 | 0.027 |
|  | Colorectal cancer | 0.001 | 0.027 |
|  | Chemokine signaling pathway | 0.001 | 0.027 |
|  | Choline metabolism in cancer | 0.001 | 0.027 |
|  | Regulation of lipolysis in adipocytes | 0.001 | 0.027 |
|  | Sphingolipid signaling pathway | 0.001 | 0.027 |
|  | Cysteine and methionine metabolism | 0.001 | 0.027 |
|  | Purine metabolism | 0.001 | 0.029 |
|  | Protein processing in endoplasmic reticulum | 0.002 | 0.030 |
|  | RNA transport | 0.002 | 0.030 |
|  | Neurotrophin signaling pathway | 0.002 | 0.030 |
|  | Endometrial cancer | 0.002 | 0.037 |
|  | mRNA surveillance pathway | 0.002 | 0.037 |
|  | MAPK signaling pathway | 0.003 | 0.037 |
| *M2 blue* | Cytokine-cytokine receptor interaction | 0.000 | 0.020 |
| *M4 yellow* | Metabolism of xenobiotics by cytochrome P450 | 0.000 | 0.000 |
|  | Glycolysis / Gluconeogenesis | 0.000 | 0.001 |
|  | Chemical carcinogenesis | 0.000 | 0.001 |
|  | Tyrosine metabolism | 0.000 | 0.001 |
|  | Metabolic pathways | 0.000 | 0.001 |
|  | Biosynthesis of amino acids | 0.000 | 0.002 |
|  | Drug metabolism | 0.000 | 0.004 |
|  | Fatty acid degradation | 0.000 | 0.010 |
| *M5 green* | Complement and coagulation cascades | 0.000 | 0.000 |
| *M6 red* | Cholesterol metabolism | 0.000 | 0.031 |
| *M8 pink* | Pancreatic secretion | 0.000 | 0.000 |
|  | Protein digestion and absorption | 0.000 | 0.000 |
|  | Salivary secretion | 0.000 | 0.026 |
|  | Neuroactive ligand-receptor interaction | 0.001 | 0.027 |

**Table S3** Enriched pathway of proteins in each module from KEGG pathway analysis. M, module.
